# Effect of Antifungal-Treated Host Macrophages on *Candida glabrata*

**DOI:** 10.1155/2021/8838444

**Published:** 2021-02-18

**Authors:** Hong-Bin Li, Na Li, Shu-Ran Wen, Ming-Yue Qiang, Zheng-Hui Yang, Tian-Xiang Dong, Yu-Ye Li, Yi-Qun Kuang

**Affiliations:** ^1^Department of Dermatology and Venereology, First Affiliated Hospital of Kunming Medical University, Kunming 650032, Yunnan, China; ^2^NHC Key Laboratory of Drug Addiction Medicine, First Affiliated Hospital of Kunming Medical University, Kunming Medical University, Kunming 650032, Yunnan, China; ^3^Scientific Research Laboratory Center, First Affiliated Hospital of Kunming Medical University, Kunming 650032, Yunnan, China

## Abstract

**Objective:**

*Candida glabrata* (*C. glabrata*) causes infections associated with severe sepsis and high mortality. This study describes the effects of micafungin (MCF), itraconazole (ICZ), and amphotericin B (AmB) on the function of macrophages during *C. glabrata* infection.

**Methods:**

RAW264.1 macrophages were treated with MCF, ICZ, or AmB and then challenged with *C. glabrata*. Cytokines from infected macrophage supernatants and the levels of superoxide dismutase (SOD) in macrophages were measured at different time points after phagocytosis.

**Results:**

The activity of SOD was significantly increased in RAW264.1 cells that phagocytized *C. glabrata* and reached a peak level at 6 hours (*P* < 0.05). ICZ and AmB did not affect the SOD activity in cells that phagocytized *C. glabrata* versus that in untreated macrophage. *C. glabrata* stimulated macrophages to secrete cytokines. Neither ICZ nor AmB affected the secretion of interleukin-6 (IL-6), interleukin-8 (IL-8), or tumor necrosis factor-*α* (TNF-*α*) by *C. glabrata*-infected macrophages. However, MCF downregulated the secretion of TNF-*α* by infected macrophages and reduced the SOD activity of *C. glabrata* compared with those in untreated controls.

**Conclusion:**

Echinocandins may increase their antifungal efficacy by altering the innate immune response of macrophages and attenuating antioxidants of this organism.

## 1. Introduction

*Candida* is generally regarded as a nonpathogenic symbiotic microbe of human mucosal tissue that occasionally causes opportunistic infections. Previous epidemiological studies from 2005 showed that the proportion of candidemia caused by *Candida glabrata* (*C. glabrata*) in public hospitals did not exceed 5–8% [1, 2]. However, with the increasing use of various iatrogenic catheters, immunosuppressive agents during organ transplantation and anticancer treatment, broad-spectrum antibiotics, and the increase in HIV infection, infections caused by *Candida* spp. have tended to be more invasive in deep organs and manifest as sepsis, fungemia, and other life-threatening conditions. Candidemia caused by *C. glabrata* accounts for 10.6% of fungemia [3].

Infection caused by *C. glabrata* has a high mortality rate of 40–70% [4]. In recent years, the success of azole antifungal agents for the control of fungal infection has been consistent with the increase in non-*Candida albicans* (*C. albicans*) infection, among which *C. glabrata* infection has increased by three times. According to the ARTEMIS Global Antifungal Monitoring Project, *C. glabrata* accounted for 10.2%–11.7% of all *Candida* spp. strains isolated from June 1997 to December 2007 [5]. A multicenter prospective survey of severe invasive *Candida* infections in China also showed that the incidence rate of *C. glabrata* in China was 12.9% [6]. It has been shown that *C. glabrata* is resistant to most antifungal drugs [7]. However, the involved mechanism is not fully understood.

Macrophages, as the first line of defense against invading pathogens, produce an oxidative stress response against pathogens while upregulating the expression of various proinflammatory cytokines and chemokines such as interleukin-6 (IL-6), interleukin-8 (IL-8), and tumor necrosis factor-*α* (TNF-*α*), thus improving the ability to kill pathogens. *C. albicans* survives in macrophages through the formation of hyphae and mycelia that penetrate the cell membrane [8–10], which causes significant toxicity to macrophages. *C. glabrata* cannot form hyphae and has no obvious toxicity to macrophages. Studies have found that *C. glabrata* does not cause significant cytotoxicity in macrophages within 48 hours but can survive in macrophages for a long time [11], indicating that *C. glabrata* can adapt to the harsh intracellular environment and evolve strategies for intracellular survival. The antioxidant system of *C. glabrata* can resist damage and can be used to avoid being killed by phagocytes. The antioxidant system of *C. glabrata* includes catalase, glutathione peroxidase, superoxide dismutase (SOD), and glutathione, and all of which are effectors of oxidative stress. SOD is very important for many pathogenic bacteria and fungi, such as *C. albicans*, *Histoplasma capsulatum* (*H. capsulatum*), and *Cryptococcus neoformans* (*C. neoformans*) [12–14]. It also plays an important role in the metabolism of *C. glabrata* and is related to the integrity of DNA and the prevention of aging [15]. In this study, we aimed to examine the role of macrophages in *C. glabrata* infection and treatment.

## 2. Materials and Methods

### 2.1. *C. glabrata* Strains

The *C. glabrata* standard strain ATCC2001 was purchased from the American Type Culture Collection (Sinozhongyuan Ltd., Beijing, China). Ten clinical strains of *C. glabrata* were from the Laboratory Department of First Affiliated Hospital of Kunming Medical University. All *C. glabrata* strains were incubated in the yeast extract-peptone-dextrose (YPD) medium (1% yeast extract (OXOID, Britain), 2% peptone (OXOID, Britain), and 2% dextrose (Beijing Second Chemical Reagent Factory, Beijing, China)) or Sabouraud agar (1% peptone, 4% glucose, 1.2% agar, and 0.025% chloramphenicol) (Bio Mérieux, Lyon, France) at 35°C.

### 2.2. Antifungal Agents

MCF (GlpBio, China), ICZ (Tixiai Chemical Industry Development Co., Ltd., Shanghai, China), and AmB (Tixiai Chemical Industry Development Co., Ltd., Shanghai, China) powder at laboratory levels were obtained and used for the preparation of stock and working solutions. The minimum inhibitory concentrations (MICs) of 11 strains against three drugs were determined by the M27-A2 method according to the National Committee for Standardization of Clinical Laboratories (NCCLS) [16].

### 2.3. Macrophages

The macrophage cell line RAW264.7 was a gift from the Department of Immunology of Kunming Medical University. The cells were cultured in a DMEM medium (Thermo Fisher, USA) with 10% FBS (Sangon Biotech, Shanghai, China) in an incubator containing 5% CO_2_ at 37°C. For each experiment, macrophages were pretreated with each antifungal drug at less than each MIC value and the plate was then incubated for 24 hours. After this pretreatment, *C. glabrata* (2 × 10^6^/mL) was added into each culture flask with 1 mL macrophages (2 × 10^6^ cells/mL) at an MOI = 1 [17]. The end points for the evaluation of each phagocytosis experiment are indicated in the Result section.

### 2.4. Determination of Cytokines and SOD Activities

To quantify cytokines, IL-6, IL-8, and TNF-*α* in supernatants from medium or yeast-stimulated RAW264.1 macrophages were harvested by centrifugation and stored at −80°C until analysis.

Infected macrophages were washed with PBS (phosphate-buffered saline, 1.06 mM KH_2_PO4, 155.17 mM NaCl, 2.97 mM Na_2_HPO_4_–7H_2_O) (Thermo Fisher, USA) to remove extracellular yeast; monolayer cells were examined under a microscope to ensure that they were intact and free of extracellular yeast. Cells were then treated with cold deionized water to breakdown and release *C. glabrata* cells that were phagocytosed. The amounts of IL-6, IL-8, and TNF-*α* were measured by specific commercial ELISA kits (Solarbio, Beijing, China) according to the manufacturer's instructions. The activity of SOD was determined by a SOD activity detection kit according to the manufacturer's instructions (Solarbio, Beijing, China).

### 2.5. Statistics

The results were obtained from at least three independent experiments and subjected to statistical analysis. Data are presented as the mean ± SD and were analyzed by ANOVA or Student's *t*-test by using Prism 8 software (GraphPad, San Diego, CA). The level of statistical significance was set at *P* < 0.05.

## 3. Results

### 3.1. General Information

A total of 10 clinical strains tested in this study were from adult patients recently diagnosed at our hospital. Among 10 infected patients, 6 patients were female. Of these, two women suffered from uterine fibroid, one from renal calculus and one from cervical carcinoma. Three *C. glabrata* strains in the male group were isolated from patients with jaundice and intestinal obstruction. The source of 10 clinical strains and detailed clinical information are shown in Table 1.

### 3.2. Drug Sensitivity of Clinical Strains

To determine the optimal concentration of each antifungal agent to examine macrophage activity, the susceptibility of 11 experimental strains to MCF, ICZ, and AmB was measured according the M27-A2 protocol of NCCLS and is shown in Table 2. Compared with the MICs of ATCC2001 for AmB (0.5 mg/L) and ICZ (0.125 mg/L), the MICs of only one strain (16K1128) for AmB and two strains (16K1132 and 17K1173) for ICZ were 4 times higher. We found that the MIC values of the four strains were at least 4-fold higher: 17K1150 (4-fold), 17K1152 (8-fold), 16K1128 (15-fold), and 17K1173 (31-fold).

### 3.3. Effects of Antifungal Agents on SOD Activity in *C. glabrata* after Phagocytosis

**Figure 1:**
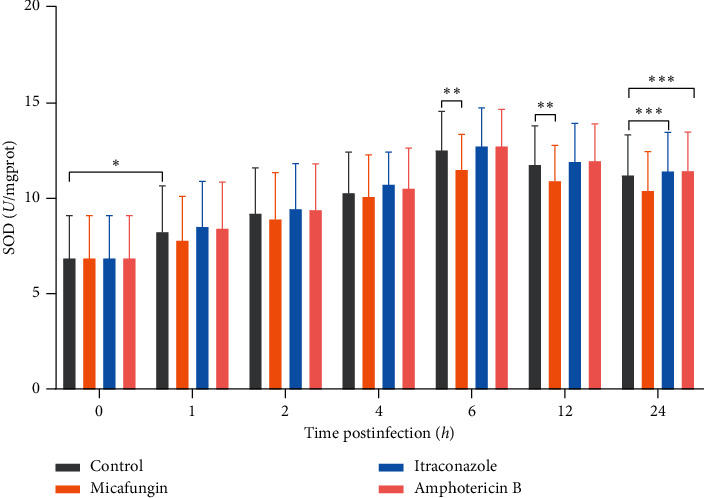
SOD activity of *C. glabrata* in macrophages pretreated with three antifungal drugs. We measured the SOD activity of macrophages treated with MCF, ICZ, and AmB. The control group was not pretreated with antifungal agents. The SOD level after 1 hour was significantly increased compared with the SOD level of nonphagocytized *C. glabrata* (0 hour) (^∗^, *P* < 0.05). The mean SOD level of fungus-infected macrophages treated with MCF was lower than that of untreated fungus-infected macrophages, especially 6–12 hours after infection (^∗∗^, *P* < 0.05). There was no significant difference in SOD levels between the untreated and ICZ- or AmB-treated fungus-infected macrophage groups (^∗∗∗^, *P* > 0.05).

*C. glabrata* tends to “hide” or “escape” from killing by phagocytes via the upregulation of the antioxidant system. SOD is at the front line of this antioxidant mechanism, and it has been adopted by many pathogenic bacteria and fungi, including *C. albicans*, *Histoplasma capsulatum* (*H. capsulatum*), and *Cryptococcus neoformans* (*C. neoformans*). In this study, we found that the SOD activity in *C. glabrata* phagocytosed by macrophages gradually increased after 1 hour and peaked at 6 hours after infection. As shown in Figure 1, compared with the SOD level of nonphagocytized *C. glabrata* (0 h), the SOD level significantly increased at 1 hour after infection (*P* < 0.05). SOD levels in *C. glabrata* cells in macrophages, with or without antifungal treatment, began to decrease at 12 hours after infection. The mean SOD level of fungus-infected macrophages treated with MCF was lower than that of untreated fungus-infected macrophages, especially at 6 and 12 hours after infection (*P* < 0.05). However, there was no significant difference in SOD levels between untreated and fungus-infected macrophages pretreated with ICZ or AmB (*P* < 0.05).

### 3.4. Cytokines Secreted by *C. glabrata*-Infected Macrophages

**Figure 2:**
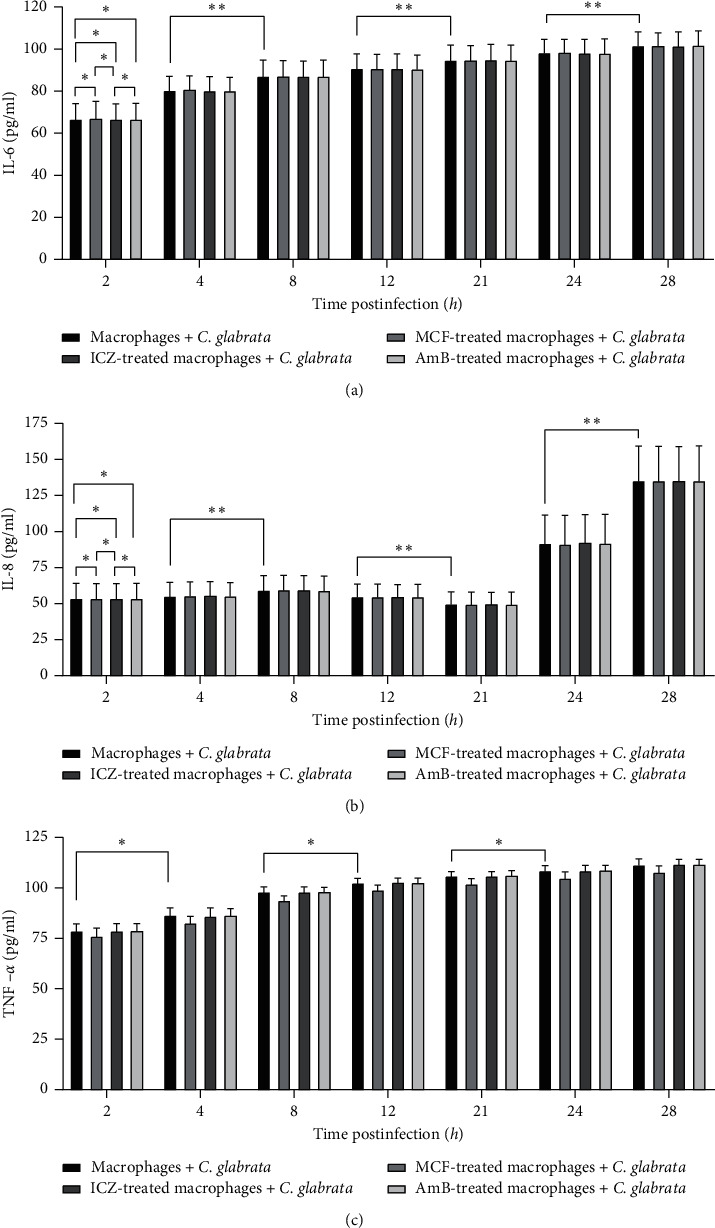
Effects of different antifungal drugs on the secretion of IL-6, IL-8, and TNF-*α* from macrophages infected by *C. glabrata*. The levels of secreted IL-6, IL-8, and TNF-*α* were determined by ELISA. IL-6 levels secreted by the four groups of macrophages were not statistically significant (^∗^, *P* > 0.05), and IL-6 levels secreted at different time points were significantly different (*F* = 6562.399, *^∗∗^*, *P* < 0.001). At all-time points, IL-6 levels gradually increased over time (a). IL-8 levels secreted by the four groups of macrophages were not statistically significant (^∗^, *P* > 0.05), and IL-8 levels secreted at different time points were statistically significant (*F* = 828.076, ^∗∗^, *P* < 0.001). At all-time points, IL-8 levels fluctuated with time (b). TNF-*α* levels secreted at different time points were statistically significant (*F* = 2237.196, ^∗^, *P* < 0.001). At all-time points, TNF-*α* levels gradually increased over time (c).

The production of IL-6, IL-8, and TNF-*α* can be induced by *C. glabrata* infection [18, 19]. However, the levels of cytokines secreted by macrophages after *C. glabrata* infection were significantly lower than those after *C. albicans* infection [20]. In this study, we found that IL-6 increased 5.5–8.4 times within 2–28 hours macrophage infection by *C. glabrata*, as shown in Figure 2(a) (*F* = 6562.399, *P* < 0.001). During the same time period, IL-8 increased 11–27 times (Figure 2(b)) (*F* = 828.076, *P* < 0.001) and TNF-*α* increased 7–10 times in infected macrophages (Figure 2(c)) (*F* = 2237.196, *P* < 0.001). Interestingly, IL-8 secretion by infected macrophages significantly increased at 24 hours after infection and further increased at 28 hours after infection, whereas IL-6 and TNF-*α* gradually increased at 2–28 hours after infection.

Similar to SOD activity, at any point in time, there was no significant difference in IL-6 and IL-8 secretion between each antifungal-treated macrophages and untreated macrophages or among the drug pretreatment groups after fungal infection (*P* > 0.05). However, MCF-treated macrophages showed a slight decrease in TNF-*α* levels within 4–28 hours after infection but were still higher than those in untreated noninfected macrophages (*t* = 20.611, *P* < 0.05).

## 4. Discussion

The innate immune system plays an important role in defending against pathogens, such as the opportunistic pathogen *C. glabrata*. Infections caused by *C. glabrata* have become common and cause high morbidity and high mortality. However, there have been fewer studies on its pathogenesis compared with that of *C. albicans* [21, 22]. Resisting macrophage killing is the first step employed by *C. albicans* to evade the immune system and cause disseminated infection. Cytokines secreted by macrophages recruit other types of immune cells to the infection site [11]. For example, the mycelium of *C. albicans* binds to tool-like receptor 2 (TLR2) on monocytes, thereby increasing the level of the anti-inflammatory cytokine IL-10 during infection [23]. Unlike *C. albicans*, blood infections caused by *C. glabrata* can persist for a long time in mice without causing high levels of inflammation, even in immunocompetent mice; *C. glabrata* cannot be eliminated after a few weeks [24]. Our data showed that the levels of IL-6, IL-8, and TNF-*α* secreted by macrophages significantly increased after infection with *C. glabrata*, although they were lower than those reported for *C. albicans* infection, which is in consistence to report in previous work [20].

The effects of antimicrobial agents on mammalian cells, especially those of the host innate immune response, have recently been noted, as patients with severe disease may depend on the complex interactions between antibiotics and their activity on pathogens and host cells [25, 26]. In this study, we selected three antifungal drugs to examine their possible side effects on macrophage function. These three antifungals target different components of fungi. ICZ and AmB target the fungal cell membrane, and MCF inhibits *β*-1,3-glucan synthesis for the cell wall [27]. We used a concentration of half the MIC value of each drug to examine its possible effects on cytokine secretion and fungal SOD activity; the results showed that there was no change in the levels of IL-6 and IL-8 in antifungal-treated macrophages within 28 hours after fungal infection. In contrast, TNF-*α* decreased slightly in macrophages treated with MCF. It was reported that cytokines such as IL-8 and TNF-*α* are released from human monocytes after exposure to *β*-1,3-glucan by *C. albicans* infection [28]. The reason for the decrease in TNF-*α* may be that *β*-1,3-glucan is insufficient due to the effect of the echinocandin on *β*-1,3-glucan synthesis or fungicidal effects. This hypothesis seems to differ from previous observations, which indicated that *C. glabrata* cells in replicative senescence in macrophages thickened their cell walls when treated with antifungal drugs [29]. The other mechanism may be mediated by hypoxia-induced *β*-glucan masking which is dependent on mitochondrial signaling and the cAMP-protein kinase pathway [30]. Since the levels of IL-8 and IL-6 in macrophages treated with MCF did not change, the possibility of decreasing secondary levels of proinflammatory cytokines secreted by macrophages induced by overexposure to *β*-1,3-glucan [31–33] is not yet clear.

Contrary to reports of the antioxidative activity of other fungi induced by ICZ or AmB [34, 35], we found that the low concentration of these two antifungal agents had no effect on the SOD activity of phagocytized *C. glabrata*. Similar to the TNF-*α* response to MCF-treated macrophages, the SOD activity of phagocytized *C. glabrata* was reduced when macrophages were pretreated with MCF. Here, the unidentified macrophage effector induced by the echinocandin or the killing effect of the echinocandin is responsible for the decrease in SOD. However, this observation highlights that echinocandins may have antifungal effects by simultaneously inhibiting cell wall formation and reducing antioxidants of *C. glabrata*.

These results support our hypothesis that echinocandins may directly affect the activity of human cells related to innate immunity. In fact, the effect of echinocandins on cytokines secreted by macrophages is related to the function of the endoplasmic reticulum [36]. However, we still lack adequate evidence to conclude that *β*-1,3-glucan from *C. glabrata* is associated with TNF-*α* production by macrophages. In addition, this study had several limitations. First, this study used only one dose of antifungal therapy. We evaluated the cytokines secreted by *C. glabrata*-infected macrophages, and the goal was to compare the difference between treatment with different antifungal drugs and untreated macrophages after treatment with only one concentration (1/2 MIC) of each drug for 24 hours. Small changes in cytokines between cells administered antifungal treatment and no treatment may have resulted from insufficient doses of each drug treatment. Second, the number of *C. glabrata* cells was not evaluated during the study. We observed that although the total content of antioxidant SOD increased at 6 hours after antifungal treatment, MCF caused a slight decrease in the SOD activity in phagocytized *C. glabrata* cells. Compared with the other two antifungal drugs and no treatment, the fungicidal effect of MCF may lead to a decrease in *C. glabrata* cells in macrophages. Third, the amount of fungal sample used in our study was very small. Further studies are required to verify the current results with a larger sample size.

The persistent presence of *C. glabrata* and the weak inflammatory response in tissues remain one of the biggest challenges in the treatment of systemic fungal infections. As we observed in this study, all clinical specimens were isolated from pus or peritoneal drainage, and none of them were drawn from the blood, again indicating the chronic progression of *C. glabrata*. In the course of fungal infection, small differences in cytokines from macrophages treated with different antifungal drugs and untreated macrophages confirmed the safety of the three antifungal drugs on the immune response against *C. glabrata*. Our results also indicate that echinocandins tend to kill fungi via cell wall exposure and interfering with the response of host macrophages.

## 5. Summary

The survival and proliferation of *C. glabrata* in macrophages is an important factor in systemic infection, which depends on the ability of these fungi to respond effectively to the stressful environment and avoid immune defenses. It has long been recognized that *C. glabrata*'s intrinsic resistance [37, 38] to oxidative stress occurs through the high expression of detoxifying enzymes (catalase and superoxide dismutase) during infection [39]. Additionally, resistance to antifungals, including high-value antifungal agents such as echinocandins, has emerged in *C. glabrata* [40, 41]. Current research is still focused on the interactions between organisms and antibacterial agents, and little is known about the immune effects of antifungals during infection. To understand the effect of antifungal drugs on *C. glabrata*-infected macrophages, we pretreated macrophage cell lines with an azole, echinocandin, and AmB for 24 hours before *C. glabrata* infection. We found out that macrophages pretreatment with ICZ and AmB had no effect on cytokine production by macrophages or antioxidant SOD activity in phagocytized *C. glabrata*. However, cytokines and the activity of SOD decreased slightly after macrophages were pretreated with MCF. This study suggests that there is a complex response to antifungal drugs in fungal cells and host defense mechanisms.

## Figures and Tables

**Table 1:** Clinical information of clinical strains.

No.	Sample number	Age	Sex	Diagnosis	Source of specimen
1	17K1156	52	F	Uterine fibroid	Uterine drainage fluid
2	J1	56	M	Jaundice	Bile
3	17K1173	42	F	Renal calculus	Peritoneal draining liquid
4	17K1152	80	M	Intestinal obstruction	Peritoneal draining liquid
5	17K1150	51	F	Uterine fibroid	Uterine drainage fluid
6	16K1132	27	M	Jaundice	Bile
7	16K1128	38	F	Cervical carcinoma	Pus
8	17S709	25	F	Candidal vaginitis	Leucorrhea
9	16S482	45	F	Candidal vaginitis	Leucorrhea
10	16S506	60	M	Deep fungal infection	Tissue block

**Table 2:** MIC (mg/L) of experimental and standard strains.

Sample number	MCF	ICZ	AmB
ATCC2001	0.032	0.125	0.500
17K1156	0.016	0.250	0.250
J1	0.064	0.125	1.000
17K1173	1.000	0.500	0.500
17K1152	0.256	0.250	1.000
17K1150	0.128	0.125	0.250
16K1132	0.032	0.500	0.500
16K1128	0.500	0.250	2.000
17S709	0.064	0.0315	0.250
16S482	0.032	0.250	0.500
16S506	0.016	0.0625	0.250

The MIC of three drugs in the same species was significantly different (*F* = 5.066, *P* < 0.05). There was no significant difference in the MIC among different strains for the same drug (*F* = 1.275, *P*=0.302).

## Data Availability

The data used to support the findings of this study are included within the article.
